# The mental hygiene movement: the birth of global mental health in India

**DOI:** 10.1017/mdh.2025.10035

**Published:** 2026-01

**Authors:** Shilpi Rajpal

**Affiliations:** Centre for Culture and the Mind, Department of English, Germanic and Romance Studies, University of Copenhagen, Copenhagen, Denmark

**Keywords:** Mental hygiene, Decolonization, Global mental health movements, Eugenics, Motherhood, Degeneration

## Abstract

Developments such as the opening of the first psychiatric outpatient clinic, the emergence of psychiatric social work, the surge of interest in psychology and psychiatry, and the tightening of notions about sexual hygiene, intersected with the rise of the mental hygiene movement in India from 1930s. There exists little to no discussion on how mental hygiene developed in the colonies. This study is the first to shed light on the lesser-known chapter of psychiatry in India. The dynamics of family, childhood, and nation-state when merged with ideas about racism, caste, and communalism were critical in the making of new nation-states like India. Moreover, the trajectory of India’s participation in international health movements, such as psychoanalysis and mental hygiene, allowed for exchange and participation. India’s participation in the mental hygiene movement allowed the growth of psy-disciplines in innumerable ways. This paper fills in a major lacuna in historical writing by providing an outline of the number of interconnected developments in the colonies, which are often sidelined. The international visibility of India also permitted India to take centre stage in many significant studies that were conducted by the World Health Organization after the Second World War.

Developments such as the opening of the first psychiatric outpatient clinic, the emergence of psychiatric social work, the surge of interest in psychology and psychiatry, and the tightening of notions about sexual hygiene, intersected with the rise of the mental hygiene movement in India from 1930s. There exists little to no discussion on how mental hygiene developed in the colonies. This study is the first to shed light on the lesser-known chapter of psychiatry in India. The dynamics of family, childhood, and nation-state when merged with ideas about racism, caste, and communalism were critical in the making of new nation-states like India. Moreover, the trajectory of India’s participation in international health movements, such as psychoanalysis and mental hygiene, allowed for exchange and participation. India’s participation in the mental hygiene movement allowed the growth of psy-disciplines in innumerable ways. This paper fills in a major lacuna in historical writing by providing an outline of the number of interconnected developments in the colonies, which are often sidelined. The international visibility of India also permitted India to take centre stage in many significant studies that were conducted by the World Health Organization after the Second World War.

Mental hygiene hypothesised that a psychiatrist was needed, much like a priest in channelling the inner self. The notions of mental health were tied to the controlling and guiding of emotions, habits, time, and energy. The selfhood was defined and managed through ‘courage’, ‘faith’, and ‘self-will’, which were regarded as cornerstones in mobilising human energy.^1^ The mental hygiene inherited concepts from moral management.^2^ The power of psychiatry had resurrected itself by aligning itself with biology and public health.^3^ This paper is largely based on an analysis of articles published in the *Quarterly Bulletin of the Indian Association for Mental Hygiene between the 1930 and 1938.* The journal is a rich source for the mental hygiene movement and provides a wealth of information on the workings of the movement in India and across the globe.^4^ The *Bulletin* is a remarkable repository of discussions, associations, and networking that allowed India to become an integral part of transnational networks of psychiatry and psychology.

Using the micro-historical method, the paper aims to expand on the characteristics and contradictions of mental hygiene in India.^5^ The Indian mental hygiene movement replicated the English model.^6^ The similarity in design and content, as well as the attempts at policymaking were often explicitly made at comparable levels. A number of articles published in the British Journal called *Mental Hygiene*, published by the National Council of Mental Hygiene, were republished with permission. However, there exists no evidence that the impetus for the movement came from Britain or that British mental hygiene movement directly supported the Indian movement. The movement was homegrown to a substantial extent, although the movement was modelled on the British one. In other words, the mental hygiene movement was an amalgam of colonial and nationalist aspirations.

The movement was intimately entwined with colonial structures, personnel, and ideas. The necessity of mental health policies and mental health infrastructure was often elaborated by British psychiatrists who worked in mental hospitals in India.^7^ The Indian movement reflected and reintegrated Indian political, social, cultural, and public health complexities. Mental hygiene in India was not nationalistic in its inspiration.^8^ Psychiatry in India reverberated with the difficulties of independently thriving as a profession, and internal struggles of Indianisation and inherent racial competition reflected in limited participation or aspirations.^9^ Owen Alexander Berkeley Hill (1879–1944) remained the most significant force who was behind the workings of mental hygiene in India. He passionately and vigorously organised and managed the movement.^10^ Hill was at the vanguard of expounding the need for policymaking at medical, educational, and penological levels. The *Bulletin* also gave space to educated members of the Indian middle class to voice their concerns and issues. Hygienists envisioned mental hygiene being integrated into institutions through state control. Unfortunately, the colonial state was not keen on the painstaking process of establishing or extending the mental health programme.^11^

The language of positive eugenics reflected and informed the historical development of the mental hygiene movement in India. The notions about ‘improvement of race’ impacted ideas about health, family, motherhood, disability, and mental illness. Historians have argued that the role of negative eugenics has been explored, while the political and social impact of positive eugenics needs to be outlined.^12^ The article, then, will delineate how positive eugenic thinking linked family life to regularising the role of motherhood and streamlining ideas about marriage, childhood, and mental deficiency in India. Positive eugenics meant transformation of living conditions for the creation of healthy future generations.^13^ The ideas about degeneration were connected to the national ideals of regeneration. Home, community, society, and nation gained new meaning, and the intent to preserve them became extremely crucial. This research on the Indian mental hygiene movement will be a significant contribution to historiography. It will broadly focus on the inception of the mental hygiene movement from the year 1929 to the movement’s decline after the journal ceased publishing in the year 1937. This is the first study to recount the dissemination of mental hygiene’s ideas and propaganda in the colonial world. The focus is only on key themes delineated in the *Bulletin* due to limited space. The first section of the paper describes the aims, aspirations, and activities of the movement in India. The second and third sections focus on how the use of psychiatric knowledge attempted to reorder the family life of Indians. Psychiatry’s merging with sexology provided the newly formed sexual morality an impetus from science to control and regulate sexual impulse for better procreation. The alteration of domestic life because of women stepping out of their homes was reiterated in psychiatry’s discourse as a threat to the sanctity of motherhood, destabilising children’s emotional well-being. The last section discusses the efforts of dealing with and handling the so-called ‘national problem’ of feeble-mindedness.

## The mental hygiene movement in colonial India

The term ‘mental hygiene’ dates back to the mid-nineteenth century. In 1893, Isaac Ray (1807–1881), founder of the American Psychiatric Association, defined it as ‘the art of preserving the mind against all incidents and influences’. Clifford W. Beers (1876–1943) attempted to commit suicide at the age of twenty-four. After his internment and recovery at a mental hospital, he wrote his autobiography entitled *A Mind That Found Itself.* His autobiography brought him immediate recognition, and he became a champion of the mental hygiene movement. He was the key figure behind the establishment of the National Committee of Mental Hygiene (NCMH) in 1909. The main objective of the NCMH was to encourage the institutional growth of the movement and to change public attitudes towards the insane and, ultimately, to educate the public to enable them to lead mentally healthy lives.^14^ Historian Gerald Grob asserted that *A Mind That Found Itself* had a comparable impact to that of *Uncle Tom’s Cabin.* ^15^ Beers, with the help of Swiss psychiatrist Adolf Meyer (1866–1950) raised funds and founded the NCMH.^16^

By the 1920s, the mental hygiene movement had become an international movement.^17^ The British counterpart of the National Council of Mental Hygiene was founded in 1922. Though influenced by the American movement, the British NCMH developed an alternative identity because of considerable state involvement from the beginning. In 1929, the Indian Association of Mental Hygiene was established, for which the British Association of Mental Hygiene was the model.^18^ There is no account of the mental hygiene movement in India, and therefore this paper will contextualise the exchange, transfer, translation, appropriation, and afterlives of these ideas and events that occurred in India.

Owen A. R. Berkeley Hill (1879–1944) was the superintendent of the European Mental Hospital at Ranchi. He was the editor in charge (1930–1937) of the *Quarterly Bulletin* and was actively involved in both the psychoanalytical movement and the mental hygiene movement. He took charge of the *Bulletin* and wrote several essays on many aspects of mental hygiene. Hill was educated in Rugby, Gottingen, and Oxford, but after joining the Indian Medical Services, he spent most of his life in India. Ashis Nandy has argued that Hill was ‘simultaneously repelled and seduced by imperial England and Brahminic India…’.^19^ Waltraud Ernst writes, ‘Hill married Karimbil Kunhimanny, who belonged to a lower caste (Tiyyan), and Berkeley-Hill had been subject to social sniggering to the extent that he was (wrongly) remembered even by Dhunjibhoy’s daughter to have married an “untouchable” or “sweeper woman” – an act detested by most Indians and considered as beyond the pale by Europeans in India.’^20^ She also noted that Tiyyan is a caste name referring to one of Kerala’s lower castes. In order to be able to marry, Kunhimanny joined the Brahmo Samaj.^21^ The act of an interracial marriage was unacceptable and would have made him an ‘outcast’.

Asylums were an integral part of colonial medicine. There exists a rich historiographical literature defining the characteristics of Indian colonial asylums. Waltraud Ernst has delineated the limited nature of colonial medicine.^22^ Historians of the subcontinent agree that there was no great confinement in the Foucauldian sense. However, James H. Mills has argued that the asylums were meant to ‘control’ and ‘reform’ the delinquent population.^23^ Nile Green maintained that the asylums played an important role in street clearing.^24^ Colonialism had an interesting fixation with the management of the transient population in the nineteenth century.^25^ The historical scholarship on psychiatry in the twentieth century remains underdeveloped. Waltraud Ernst has also focused on the growth of the Indian Mental Hospital in Ranchi in the twentieth century.^26^ The psychoanalytic movement has also received much-needed attention from anthropologists and historians.^27^ The mental hygiene movement in India has been overlooked by historians.

The aims of the Indian Association of Mental Hygiene were to ‘combat prevailing ignorance, encourage scientific study and to improve the psychological environment and develop programmes related to the mental health of the community’.^28^ The ideas of preservation were central to mental hygiene and were allied to economic preservation against spending on mental health. The hygienists’ ideals evaded the state’s responsibilities by attributing ‘guilt’ and emotional and financial ‘burden’ to the patients. These notions fitted well with the workings of colonialism. The aim was also to ensure propaganda work by holding regular meetings and encouraging members and experts to present their research.

The dissemination of the mental hygiene movement’s ideas to the public was attempted through establishing a popular lecture series. Another core objective was to create a central body to which difficult cases encountered in penological, scholastic, magisterial, and other work may be referred for advice. The Calcutta branch remained the most vigorous in defining the movement’s character in South Asia. The editorial note of the *Bulletin* in 1931 mentioned that ‘[d]uring the past twelve months some of the foremost citizens of Calcutta founded the Calcutta Branch of the Indian Association of Mental Hygiene with the Hon’ble Sir George Rankin, Chief Justice of Bengal, as its President and the Hon’ble lady as its Patron’.^29^ This branch of the Indian Association for Mental Hygiene regularly organised classes discussing ‘the study of elementary principles for Mental Hygiene’.^30^ Dr. B. C. Ghosh played an important role and conducted classes on a regular basis. The meetings and classes were held at 25 Chowringhee Lane through the courtesy of the authority of the Y.M.C.A.^31^

The aim of the Calcutta Branch was to establish clinics for children and to build a home for ‘mentally defective’ children.^32^ The movement in India organised Health Week Exhibitions and distributed charts, diagrams, and leaflets.^33^ It was held in Calcutta with the help of the Department of Psychology, University of Calcutta. The *Indian Journal of Psychology* gave a detailed description of the work performed by the Department of Psychology in collaboration with the Indian Association of Mental Hygiene:

By public lectures and demonstrations, radio talks and popular English and Vernacular articles in monthly magazines and other periodicals both the staff and students of the department have sought to impress upon all the necessity of looking at the problems of life from a fresh angle and to seek new remedies for and new methods of prevention of mental maladjustments. The labours of the department have not got in vain. The regular stream of visitors that call on the staff of the department of expert advice regarding themselves or their children’s aptitudes, intelligences, etc., easily bear out the above statement. The department is frequently consulted by such organisations as the Borstal, the Juvenile Offenders’ Court, the Refuge and other similar institutions. The Police even did not hesitate on one occasion to send three suspected thieves for being psychologically tested. The department is always ready to help anyone who is in need of its help; and with this object in view, it has undertaken to publish periodically pamphlets dealing with the problems of mental life in general and the mental health of the children in particular.^34^

The hygienists’ propaganda penetrated the hospital, the school, the prison, and the courtroom alike. The making of citizenship was accomplished at the levels of both the educated and uneducated masses. The educated middle class was scientifically informed by teachers, professors, and doctors who believed in ‘positive eugenics’. This is also the time when the journal *Marriage Hygiene* was first published in Bombay. The journal advertised in the *Quarterly Bulletin*, wherein the Society for the Study and Promotion of Family Hygiene, including Sex Hygiene, emphasised the merits of sexology and its relationship to other sciences. *Marriage Hygiene* aimed to improve the future of races by advising in terms of psychological, medical, social, legal, and other matters.^35^ Information on contraception, sex disorders, sex education, family life, venereal diseases, sterilisation, and child guidance was disseminated. The eugenic agendas of the *Quarterly Bulletin* and *Marriage Hygiene* overlapped, allowing the educated and uneducated masses to be fuelled and guided by impulses of nationalist and communal anxieties, and as a result, they developed their own version of eugenicist ideas, which were based on caste and communal lines.^36^

The connections between mental hygiene, child welfare, and child guidance movements are crucial to understand the conceptualisation of nation-building reforms that were often couched in the language of medicine and science. The Indian Association aimed to establish children’s psychological clinics covering various regions of India. The aim remained unfulfilled as the Indian Association had limited outreach. A number of Children’s Aid Societies existed in India from 1890s. The idea was to offer ‘protection’ and ‘care’ to children and all those who lacked any adult protection and guidance. The discussion in regard to the Society for the Protection of Children in India was often outlined in great detail regarding protection and treatment of children in danger.^37^ The *Quarterly Bulletin* also combined the Society for the Protection of Children’s principles and acted as an informal newsletter publishing details of the Society. In other words, children were regarded as future citizens and as potential assets that required significant investments in shaping their minds and bodies. Thus, the management of juvenile delinquency was a foundational aspect of mental hygiene since the prevention of crime and the formation of the youth’s character were considered pertinent to the nation’s life. Mr. H.C. Bivar, Session Judge of Behrampore, wrote ‘[t]he youth of one generation were the citizens of the next, and every youth saved from a career of crime means one more honest citizen in the next generation’.^38^

Borstal institutions in India were an extension of the British Borstal Association, wherein the reformatories were meant to provide care to the juvenile delinquent. These institutions were designed to train these young offenders to work and to save them from crime.^39^ The Borstal Association of Bengal operated on similar principles. The boys were on license, which meant they were supposed to work and not leave without formal permission.^40^ Matthew Smith, Vicky Long, Oonagh Walsh, and Despo Kritsotaki have argued that ‘[t]he emphasis on prevention accelerated during and after the Second World War in response to domestic and military pressures. On the home front in Britain, the mass evacuation of children drew attention to children’s psychological development and ways of protecting their mental health’.^41^ In other words, mental hygienists were occupied with the notions of ‘saving childhood’ and ‘youth’s character building’ as an underlying agenda of mental/racial/moral upliftment of the nation-state.

The mental hygiene movement, through its day-to-day activities, endeavoured to propagate and penetrate into the community life of the masses. The clinic established in the Carmichael Medical College was an important space for disseminating knowledge and training. The report on the working of the psychological clinic at Carmichael Medical College, Belgachia, for the year 1934 mentioned^42^

The Clinic remains open from 8 A.M. to 10 A.M. on Tuesday and Thursdays. Patients come to the clinic from Calcutta and its suburbs as well as from outside stations. The total number of cases treated up to the present time is 307. During the year under review 205 cases were attended to…47 of the Carmichael Medical College in batches of five and 11 students from the Department of Experimental Psychology, University of Calcutta, attended the Clinic where clinical lectures and demonstrations were held regularly. Several teachers from Lahore and other Universities as well as a few private medical practitioners attended the Clinic for training.

The enthusiasm to learn from the mental hygiene movement was visible in school teachers, college lecturers, lawyers, judges, physicians, and administrators. Norman J. Pacheco, the Superintendent of Ranchi Mental Hospital, wrote that ‘Mental Hygiene, like [m]edicine is an art rather than a science and its technique is derived from the techniques of medicine, psychiatry, psychology, education and social welfare work.’^43^

Berkeley Hill regularly delivered lectures on various aspects of mental hygiene. He also lectured in St Paul’s School in Darjeeling on themes like child psychology, the relationship between modern psychiatry and the law, parents and teachers, among others. Some of these lectures were presided over by Sir Jadunath Sarkar. Sarkar was a distinguished historian and vice chancellor of the University of Calcutta (1926–1928).^44^ St Paul’s School was a renowned private boarding school for boys that catered to the elitist gentry of the colonial era. Childhood remained a single-minded obsession of the hygienists. The moulding of mind and character from early childhood was intrinsic to the making of mental hygiene in India as elsewhere in the world. Hill vigorously contributed to the *Bulletin* and remained the sole editor-in-charge of it during his lifetime. In 1937 Hill issued an appeal, stating that ‘for the welfare of the Indian Association of Mental Hygiene, it was time for a younger editor, more in touch with current events than he was, to assume the editorship of the Bulletin’.^45^ Banarasi Das offered to take over the editorship of the *Bulletin*, but then the journal ceased to publish. Hill’s energetic drive and dominant personality restrained the movement as he actively involved his friends and kept rivals away from the movement.^46^ Hill mentioned that ‘the Bulletin depends too much on the contributions from persons outside India, which, however important, do not arouse the same interest as would contributions from persons belonging to the country’.^47^

Waltraud Ernst has elaborated on the latent rivalry between Hill and Major J.E. Dhunjibhoy, who was the superintendent of Ranchi Indian Mental Hospital. She argued that the European Ranchi Mental Hospital gained more popularity and favour from the government than the Ranchi Indian Mental Hospital. There existed two mental hospitals for housing European and Indians could be housed separately. A glimpse of their rivalry was visible as Dhunjibhoy declined to participate actively in the mental hygiene movement. In an editorial, Hill bluntly criticised Dhunjibhoy and publicly demeaned him by stating, ‘[w]e take this opportunity of recording our disappointment at being able to get Major J. E. Dhunjibhoy, Superintendent of Ranchi Indian Mental Hospital to join I.A.M.H. It appears to us to constitute almost an obligation in a man of his position to associate himself with the only “Mental Hygiene Movement of India.”^48^ These structural inequalities were inextricably linked to race-class-status symbolism that was attached to the hygienist movement. Berkeley Hill also widely practised psychoanalysis. Ernst also argued that ‘[p]sychoanalysis was highly fashionable during the interwar period, attracting much attention on the part of elite European and Indian society in Calcutta’.^49^ The psychoanalytic movement and the mental hygiene movement attracted the educated elite group of people. These were middle-class, educated, and scientific-minded gentry. In other words, the hygienists were a small intellectual group mostly constituted of the colonial educated elite that formed its core.

The movement remained confined to a small stratum of people and failed to arouse the interest of the masses. Hill lamented that mental hygiene had not received its due status in medicine. In an editorial of January 1937, he mentioned that ‘the Prince of Wales Medical College, Patna, recently brought out a new syllabus of training for its students. The syllabus contains no mention of training in either psychiatry or “mental hygiene”’.^50^ He also concluded that ‘so long as the medical profession in India, official and unofficial, shows such apathy to psychiatry, mental hygiene and prophylaxis…it is not be expected of the laity of realise the full importance of human health and happiness’.^51^ The colonial state was not interested in reforming medical systems and introducing mental hygiene. The country was witnessing serious anticolonial struggles during the period. The journal reflects a mindset and a moment in the history of psychiatry. The contributions published in the journal from Indians were few, but an extensive list of renowned people who were members of the Indian Association of Mental Hygiene demonstrates curiosity in and the popularity of the movement. The movement failed to penetrate deep into society, but perceptions about sexual hygiene and mental health percolated through various mediums and vernaculars which have been discussed elsewhere.^52^

## Family life

Sick nursing, maternity work, child welfare and social work are some of the channels of sublimation for women: community service, hard physical labour, games and sports provide paths for men.^53^

Banarasi Das

In the twentieth century, psychiatrists influenced by notions of global domesticity played an important role in the demarcation of gendered roles. Banarasi Das was the editor in charge of the *Quarterly Bulletin* after Berkeley Hill’s retirement in 1937. He was also the superintendent in charge of Agra Mental Hospital. Das provided a psychiatric survey of life in which he addressed and discussed a psychiatrist’s approach of ‘how to live a balanced life’. He delineated various stages and defines roles that are suited for the sublimation of genders.^54^ The mental hygiene movement was a didactic programme of teaching the public the ways of healthy living. This prescriptive way often meant to offer solutions to problems concerning modern life. The father was a lawmaker, whereas the mother a caretaker. These norms had Victorian resonance. Rules for mental life were established and were often based on a gendered division of tasks. P.G.G. Unnithan argued for an effective working of the mental hygiene ‘[e]ducation of fathers, mothers and women who were potential home-makers, on the importance and essentials of healthy habit training involving proper emotional control in children’.^55^ The fear of ‘unruly’ women who could become ‘disobedient wives’ and ‘wicked mothers’ and, thus, were incapable of being ideal mothers/homemakers is pertinent in psychiatric texts. Berkeley Hill while describing an ideal motherhood, remarked ‘[a]nother type of maternal horror, is the woman who is ashamed of being a woman the tom-boy grown up. It is she who tells her daughters never to marry and never to trust a man. It is she, jealous of the male, who turns away from the little son who runs to her naked, saying, “Fie – cover it up”’.^56^ Women’s conception as ‘maternal’ and ‘homely’ was reinvented in the mental hygiene literature. A. Helen Boyle (1869–1957), a prominent British psychiatrist whose work was reprinted in the Indian *Quarterly Bulletin*, explained, ‘A crop of healthy, hearty and intelligent children is a good one and the only one that concerns us to-day.’^57^ She expected women to leave their work after giving birth to children. Women should spend their time carefully. She explained, ‘[f]amily is worth saving. No other plan offers such a chance of individuality and such a variety of environment’.^58^

The political, social, sexual, and psychic lives of Indians were under tight scrutiny. Indians were blamed for being ‘backward’ and ‘regressive’, and their sexual and marital practices were attacked at the transnational level.^59^ Katherine Mayo was the American journalist whose book *Mother India*, published in 1927, attacked Indian religion and politics, questioning self-governance and justifying imperial rule in India.^60^ Mayo’s work was published in 1927, creating controversy and generating ripples on the international stage. The essential case was that of the Indian child bride whose piteous situation necessitated protection and intervention from the civilised world. The representative image of the child bride and degenerate Hindus entrenched the colonial control over the ‘political’ and the ‘social’ spheres. The book was translated into German, French, Italian, Danish, Dutch, Swedish, and Hebrew.^61^ What Mayo argued had become a ‘character statement’ about the ‘degenerate’ Hindu and therefore was taken as an attack on the then burgeoning Hindu nationalism. In other words, Mayo’s book generated responses from various national leaders, including Mahatma Gandhi, who called it ‘Drain Inspector’s Report’.^62^ Mrinalini Sinha argued this controversy became ‘a byword for imperial propaganda’,^63^ and was crucial for forming rhetoric on ‘growing economic and political muscle of the US on the world stage’.^64^ She also pointed out that ‘the controversy had created an opening for an unprecedented development: the first universally applicable law regulating marriage for all communities in British India’.^65^ Ishita Pande asserts that Mayo’s book coincided with ‘India’s coming of age during the interwar years, one must pay greater attention to the sex life of the nation as it was debated not only by Mayo in her provocative book but also by various members of the newly reformed Legislative Assembly who seized upon child marriage to debate India’s past, present, and future.’^66^

‘Home’ was the microcosm of the nation, and family life was vital for the stability of the nation-state. The family had to be preserved by the new legal framework and pedagogies of sexology that overlapped psychology and psychiatry. Sexology became a guiding light to reform Indian sexual practices. Sexual hygiene at best remained an ambiguous term which was used to envision ‘better procreation as a better future’.^67^ Berkeley Hill, in his essay on ‘Sexual Hygiene’, discusses the need for modernising marriage, eliminating taboos, and revising conventional sexual morality. He quoted men such as Kraft-Ebing, Magnus Hirschfeld, Sigmund Freud, H. G. Wells, and Bernard Russell and argued that ‘only a scientific study can solve the problems related to sex’.^68^ He called for ‘the celebrated campaign for the rehabilitation of the unmarried mother and the protection of the illegitimate child’ in France.^69^ He campaigned in favour of birth control. Hill described Sigmund Freud’s ideas on neuroses and sexual abstinence and further remarked that ‘[m]any people who pride themselves upon having remained abstinent, have only managed this with the help of masturbation…[i]t cannot, in other words, help to build up energetic, independent men of character, original thinkers, bold advocates of freedom and reform, but leads rather to produce good weaklings who become in time completely merged in the great mass of mediocrities’.^70^ Masturbation was linked to degeneration in both the Western and the vernacular medical literature.^71^ Sigmund Freud’s famous hypothesis on hysteria and masturbation was well known. While Freudian notions about masturbation underwent change by the turn of the century,^72^ masturbation in India continued to be known as a disease *par excellence.* ^73^

Berkeley Hill, in another article titled ‘Autoeroticism’, dealt in detail with the side effects of masturbation. He referred to Havelock Ellis’ broader definition of autoeroticism as ‘any manifestation of the sexual impulse towards own’s self’.^74^ His entire essay focusses on masturbation and its effects on men and women. Hill asserts that ‘the solitary sex’ had far more serious effects on men than women. He regarded women as mostly ‘ignorant’ and not ‘really aware of what they are doing’.^75^ He denied women having any agency and commented, ‘[t]he fact that the genital organs of women are not “external”, in the sense they are in men, may play a considerable part in this apparent immunity of women from the feeling of shame and degradation which is so common amongst boys and men. He exclaimed, ‘[m]asturbation could drive a man to suicide.’^76^ He was aware of the changed views but stated that ‘[i]t was discovered that only very rarely could any disorder be directly traced to the practice of masturbation and that when any serious consequences could be proven, they were not due to the actual practice of masturbation but to the feeling of shame, guilt and terror to the habit gave rise’.^77^ He abided by Freudian ideas about masturbation and its ill effects. George J. Makari pointed out that ‘sometime between 1901 and 1905, Freud’s understanding of masturbation shifted along these lines…such normal infantile masturbation was now only pathological if it continued into latency’.^78^ While Freud was disenchanted with his earlier hypothesis of childhood that masturbation was dangerous, he continued to regard adult masturbation as harmful.

Opinions regarding ‘the vices of solitary sex’ were widely known to the public in the context of colonial India. The nation feared degeneration and felt emasculated under British rule and turned towards their own version of ‘better procreation’.^79^ The imagined threat of Muslims breeding much faster than Hindus led to widespread publishing of pamphlets in vernacular languages concerning saving youth through the practices of celibacy/*Brahmcharya.* ^80^ Ishita Pande discusses Yashoda Devi, a female Ayurvedic doctor in the 1920s, who wrote many tracts in Hindi describing correct sexual practices to men but mostly women. Devi regarded ‘[m]asturbation was pronounced most dangerous, and the reason why many young men entered the conjugal state exhausted’.^81^ She wrote several books on the ideal role of husband/wife. Pande asserts that ‘Yashoda Devi claimed to treat sexuality according to the scientific principles of Ayurveda. Like other contemporary authors who embraced the rhetoric of science, she aligned sexual hygiene with eugenic reproduction and provided advice on how often and when to have sex and how to conceive and raise a healthy child’.^82^

The mental hygienist’s emphasis on childhood as the most crucial stage of development was replaced in the Hindi medical literature by youth as an age of precarious dilemmas. Semen was regarded as life essence or sacred nectar. Young men were warned against the dangers of masturbation. Charu Gupta argues, ‘[B]rahmacharya thus became a building block for claims to social and political power, cultural identity and a “scientific” way of life.’^83^ ‘Saving semen’ was equated to ‘saving nation’. Sarah Hodges noted that ‘the Indian conception of eugenics was formulated around nation-building and was split between the two opposite concerns “saving semen” and “the use of birth control”’.^84^ These concerns often were related to heightened communal tensions, and it was also believed that the use of birth control helped in ‘better procreation’, and ‘saving semen’ aided in the preservation of youth. She further asserts that ‘…eugenicists asserted that Indians could manage their own reproduction and in so doing breed a better India. Some connected individual reproductive self-governance to demonstrating fitness for formal political autonomy’.^85^ Indian eugenicists believed that the widespread use of birth control would help in eliminating poverty.^86^ Positive eugenics was a result of social Darwinism and had a widespread appeal to policy makers, medical scientists, social reformers, and scientists. Erika Dyck pointed out that ‘In 1883 Francis Galton, cousin of Charles Darwin, coined the term “eugenics” to mean “nobility in birth.” The possibilities captivated reform-minded enthusiasts who sought scientific solutions to a range of problems associated with urbanisation, disease, poverty, moral degeneration, immigration, and race suicide’.^87^ Dowbiggin has argued in the context of America that ‘[i]t was the age of “Progressivism,” stretching roughly from the 1890s to the New Deal of the 1930s, years when Americans and Canadians were inspired by the dream of reform. During this period, eugenics emerged as a quintessentially progressive reform movement. Issues of reproduction, heredity, public health, and racial anthropology gripped the public’s imagination, and the word “eugenics” seemed to be on the lips of countless prominent Americans’.^88^

The ideas about sexology also had a direct correlation with eugenics. The emergence of sexology as a unique ‘science’ of procreation had an influence on both sides of the spectrum. The narrow ideals of biological determinism were revamped in scientific terms. The mental hygiene movement, with the help of psychiatrists, psychologists, and psychoanalysts, fabricated maternal ideals as a true basis of family life. These views were refurbished in the colonial context and were aligned with the needs of the nation and nationalism as they became an exigent demand for ‘citizenship’. Ishita Pande elaborates on ‘[t]he texts of global/Hindu sexology helped naturalise age (and the law’s temporality) while also representing reproductive temporality as an “ancient” Hindu value. While looking to the future to connect sexual norms to national stability, Hindu sexology cast reproductive temporality back to a Hindu past, to potent effect for the future nation’.^89^ The nation needed sons to valourise *Bharat Mata/*Mother India, who was shackled in the chains of colonialism. Ayurvedic pasts and modern medicine helped to conjure up the domesticated wife, the disciplined husbands, and the sacrificial children.

## Mother and the child

Indeed, it is now a well-established fact that the most serious disturbances in mental life are undoubteably associated with severe deprivations in the suckling period.^90^

Owen A. Berkeley Hill

Motherhood and childhood also experienced medicalisation by the beginning of the twentieth century.

Motherhood was constructed in the light of the new paradigm called ‘Mothercraft’. Advice on breastfeeding and infant care was influenced by New Zealand physician Truby King, who advocated medical supervision by nurses, doctors and, above all, clocks. Feeding by the clock became indispensable to infant care. King worked as the medical superintendent of the mental hospital of Seacliff Mental Asylum in New Zealand, and was deeply concerned with racial degeneration. The belief was that the lack of regularity can cause imbecility and epilepsy. Ranjana Saha has argued that ‘child welfare propaganda frequently deployed the widely “pervasive doctrine of maternal ignorance” to demand education of “ignorant” mothers often blamed as “houses of illness”’.^91^ This propaganda was highly racial and colonial in nature. Saha asserts the ‘“women’s question” as crucial to colonial “civilising missions”, nationalism and community-building, and even preservation of the imperial race in India. This, in turn, is intimately connected with the fact that maternal and child welfare propaganda was taken up by quasi-governmental organisations set up by the vicereines of India’.^92^ With the rise of scientific motherhood in the metropolis, Indian mothers were targeted as ignorant and incapable of taking care of their children. This propaganda was connected to the colonising ‘civilising mission’ and the ‘self-civilising mission’ of the middle class, who were quick to absorb these modernising habits.^93^

The framework of the child welfare movement imposed the concepts of regularity, and Truby King’s standards were established. Dr Ruth Young (1884–1983) was the director of the Maternity and Child Welfare Bureau of the Indian Red Cross Society. Young explained that ‘the mental hygiene for the preschool child is the planning of his so that these successive stages will be passed through the minimum of conflict…’.^94^ The mother’s centrality in the infant’s life was often equated with the term ‘parent’. Young pointed out that ‘one might almost say that everything comes back to the mother because the regulation of their environment falls on her.^95^ She elaborates…[r]egular sleep, regular bowel function, regular bathing times. Doctors know that these things make for bodily health, but psychologists have discovered that they also make mental health…the maternal technique resides in the mother’s power of aiding these carefully timed arrangements’.^96^ In regard to the invention of scientific motherhood, Apple has argued in *Perfect Motherhood* that ‘the image of the educated mother now comes to the fore, the mother who utilizes modern science and medicine to inform her childcare practices, the mother who heeds the advice of experts. As one 1938 advertisement promised, ‘[a]dd science to love and be “a perfect mother”’.^97^ Berkeley Hill while discussing the role of parents and surrogates, elaborates ‘[n]ow although a father can, and frequently does, commit a variety of errors in his relationship to his children, it is the mother as a rule who exerts the stronger influence upon the development of her offspring so that whenever we meet a defect in character or temperament of a child, it is the mother we should first turn to in our search for the cause of it’.^98^

In a detailed article on early social development, Berkeley Hill elaborates his views on infant care. He discussed ideas about feeding and weaning, thumb sucking, cleanliness, and sexual behaviour.^99^ Influenced by Susan Isaacs (1885–1948) Hill takes a middle path in terms of the necessity of regulations. Susan Isaacs was an important theorist and psychoanalyst who was known to develop a sensitive approach to a baby’s need and did not believe in the clock’s regulations.^100^ Hill argued, taking a cue from Isaacs’ book, that suckling should be considered as the nucleus of the baby’s life.^101^ In other words, weaning meant a deprivation of mother’s love. At the same time, thumb-sucking was regarded as the child’s intense need to gratify. Berkeley Hill points out that the forceful deprivation of thumb-sucking might throw the child into ‘a state of over-whelming helpless exasperation, which must have a worse effect on him than thumb-sucking itself’.^102^ He also remarked ‘to avoid demanding too high a standard of cleanliness’.^103^ Ignoring a child’s screaming was considered unnecessary harshness, which might cause an infant ‘a deep emotional conflict and overwhelming anxieties’.^104^ In contrast to Truby King’s theories which had gained widespread popularity in the 1920s in India, Hill familiarised the people with theories that were considered novel.

According to Hill, children’s sexual curiosity might impede their development for the rest of their lives if it is not satisfied. The simple questions, like ‘where do babies come from’?^105^ can cause them obsessive anxiety. He asserted that ‘…that children’s minds are harnessed, impeded and sometimes permanently undermined by internal conflicts of one sort or another is a matter beyond dispute’.^106^ Susan Isaacs, also known by her pseudonym Ursula Wise which she used to write a weekly parenting column, ‘showed an unremitting understanding of both the child and parent while dealing with the majority of concerns’.^107^ Hill culled ideas from Isaacs’ *Social Development in Young Children* and emphasised ‘the child-derived satisfaction from the manner in which questions of this sort are answered than the information supplied…’.^108^ He remarked that sexual questions should not be prohibited, as it will leave the child to battle these questions alone. It will be no exaggeration to state that the question of early child development centred around the mother and child. In other words, there was no duty that was assigned to the father. Hill claimed that the fact remains that ‘these problems are the very pith and essence of mental hygiene in so far without a thorough understanding of them…the yearly toll of insanity and crime will continue to tax the resources of our asylums, prisons and reformatories’.^109^ K. Clement-Jones, who was the tutor to the tutor to the sons of the sixth Nizam of Hyderabad, Mahboob Ali Khan, refers to the broader term parents and teachers’ duty in answering the children’s questions pertaining to sex. However, he warns against the ‘ignorant servants, who invest the same with prurience, most harmful to the child. Ayahs and bearers, in continual attendance on children in India, unwittingly give them superstition and unwholesome views on life’.^110^ The widespread fear of the ignorant ayah or maid was pertinent to maintaining white racial supremacy. The maid’s service was crucial for the children’s upbringing, but these children should be guarded against their polluting thoughts.

The centrality given to the mother became a complex issue in the writings of mental hygienists. H. Steadman remarked that ‘the mother, who from the first has supplied the boy’s needs, and has become his first and greatest love, is clung to for protection against the outer world. The child feels his dependence upon the mother, and resents the father’s claims upon her attention’.^111^ The links between child welfare and the child guidance movement emphasised the tremendous centrality of the mother in the child’s life. Kathleen W. Jones has argued that in the context of the United States ‘[b]y 1940 child guidance was synonymous with mother-blaming’.^112^ In other words, child guidance attacked mothers for the behavioural problems of their children. Child welfare, child guidance, and scientific motherhood were intertwined in transnational ideals of global domesticity. Steadman asserted ‘[i]n later life the hated father is identified with the schoolmaster, and later still with the employer, the state, religion, and all forms of authority. The subconscious impulse springing from the childish complex may cause a lifetime of resistance to authority, and possibly crime’.^113^ The mental hygiene movement was a cradle for the child guidance movement. Child guidance was focused on the ‘problem-child’ who could only be rescued from becoming a ‘hardened criminal’ by following the principles of mental hygiene. Jones argues that ‘the team of psychiatric, psychological, and social work professionals trained to work together to evaluate and treat problem children; and a critique of parental responsibility for troublesome behaviour that was the wellspring of modern “mother-blaming”’.^114^ The rise of scientific motherhood emerged with the child welfare propaganda, which attacked traditional maternal practices as lacking scientific values. This propaganda was entangled with ideals of domesticity, shackling women into household chores, and allowing fathers to act as disciplinarians to not just the children but also their wives.

The idea of correction of the ‘troublesome’ was fundamental to the actual working programme of the mental hygiene movement in India. The main feature for an effective programme included an ‘[e]stablishment of Child Guidance Clinics for the correction of abnormal behaviour in children, like stealing, delinquency, shyness, timidity etc.’^115^ In the United States, Laura D. Hirshbein asserted that ‘the concept of juvenile delinquency has been fluid, contingent and used in wildly different ways depending on the expectations for children of any particular generation, as well as the race and class of the child in question’.^116^ The concept of juvenile delinquency in India was rooted in notions of class and race. The movement in India was to an extent popular amongst the ‘whites’ and the emerging middle class. The middle class with their self-fashioning of an ‘educated/modern’ class in India were interlocutors of the movement. This is to argue that juvenile delinquency targeted adolescents from poorer classes and lower castes. A further investigation into the category of ‘problematic’ adolescent or juvenile delinquent would help reveal caste/class biases that were shaped by colonialism but also were consciously imbibed into the making of citizens.

In India, the Child Guidance Clinic of the Sir Dorabji Tata Graduate School of Social Work was established in 1937. The clinic was not a part of the Indian Association of Mental Hygiene. A report on the clinic mentioned that it would address ‘the scientific study and treatment of children suffering from various behaviour disorders such as unmanageableness, stealing, lying, truancy, sex offences, violence, destructiveness; personality disorders such as obstinacy, shyness, sensitiveness, moodiness, depression, fears, nervousness, day dreaming; habit disorders such as bed wetting…’.^117^ John Stewart has argued, ‘[c]hild guidance thus developed in an era where “normalcy” seemed under constant threat and instability the usual state of affairs. Social, political and economic problems had created their own maladjustments, while new forms of knowledge in fields as diverse as psychology and physics contributed to the unsettling nature of modernity’.^118^ Stewart also mentioned that in the British context, child guidance emerged after the First World War. In India, the child guidance movement grew much later. It was developed under the aegis of the Sir Dorabji Tata Institute of Social Sciences, allowing it to grow in collaboration with psychiatric social work, paediatrics, and psychology. The movement developed within the progressive philanthropic framework which remained limited, and so was the backing of the state for child guidance in India.

## The problem of the feeble-minded

Even the lepers have their lazarettos and as a result of concentrated research the world over their treatment has been improved, their cure facilitated. Are these defectives to be considered worse than lepers and are we to continue shirking our duties of them?^119^

J.N.J. Pacheco

The psychiatric infrastructure was well established by the twentieth century, allowing the reification of psychiatrists to act as a professional cohort. J.N.J. Pacheco, the superintendent of Ranchi Mental Hospital, in an essay entitled ‘A Plea for the Control of the Mentally Defective Population in India’ makes a desperate plea to create provisions for the mentally deficient. He asserts, ‘[w]e do not know what our defective population is to-day…[i]f the mean incidence is 8 per 1000 of the population in an advanced country like England I feel it might be higher in the East’.^120^ The fear was that degeneration was increasing amongst the ‘natives’ who were regarded as ‘inferior’, ‘backward’, and ‘ignorant’. The enumeration of feeble-minded was not accounted for in the census report of 1921. Pacheco argued that the mental deficiency should be regarded as the ‘national problem’. He urged the state to pass a Mental Defectives Act similar to that in England. Mark Jackson argued that in England ‘the Mental Deficiency Act of 1913 and the Elementary Education (Defective and Epileptic Children) Act of 1914, which together obliged local authorities to provide special facilities for the care and control of defective children and adults, were not only strongly influenced by medical models of mental deficiency but also in turn generated a discursive site for the consolidation of those models’.^121^ India did not have any formal legislation regarding the care of mentally defectives. The absence of any institutional policy on the part of the government was regarded as sheer neglect by mental hygienists such as Hill and Pacheco. The feeble-minded were considered to be dangerous and therefore should be segregated by the mental hygienists.^122^ Jackson also pointed out that ‘[d]uring the long turn of the nineteenth and twentieth centuries, mild forms of mental deficiency were reconceptualised as pathological and as dangerous than as severe forms of deficiency’.^123^

The growing concern over the increase in the numbers of the ‘unfit’ was predominant in the so-called progressive countries. Kalidas Bhattacharya’s statistical account, enumerated in 1938, registers a similar urgency as Pacheco’s resolve to ascertain the numerical strength of the feeble-minded in India. He remarked that ‘[t]he abnormal growth of this number is always a menace to the society’.^124^ Segregation was viewed as the way forward since ‘the society needs to be protected’ from the ‘unfits’. The ideological basis of segregation was the belief that the feeble-minded were responsible for economic impoverishment. This control of their reproduction was done through policies related to the segregation of sexes and the establishment of colonies.^125^ In England, several colonies were set up providing forced employment for the ‘defectives’, which was seen as a measure to deal with unemployment. Work, therapy, and segregation were considered to be a solution. The other significant means of dealing with the mentally defective was voluntary sterilisation. The question of voluntary sterilisation was widely debated in the West. Matthew Thompson has pointed out that ‘…the British voluntary sterilisation campaign opposed castration or sterilisation as a punishment, since either would stigmatize what was hoped to be a voluntary measure and would not serve a specifically medical or eugenic role’.^126^ Owen Berkeley Hill similarly asserted that ‘[s]terilisation is not a punishment for anyone; it is a protection for all concerned and a safeguard for prosperity. Most operations therefore will be voluntary. Only a few may be compulsory’.^127^ Hill offered a financial and emotional consideration to appeal ‘the economic burden represented by the care of these mental defectives and insane, as well as their economic loss through their withdrawal from production, total nearly a billion dollars a year. But this is a small affair compared to the injustice to the defective child whose birth is permitted, the heartache of the families, and the misery spread through the community’.^128^ The question about preservation of the mind was conspicuously linked to the economic burden of the ‘unfits’. The hygienist’s concerns were in reality political and economic propaganda that was couched in the language and concepts of conservation and care. The movement was short-lived as it failed in its projection of an inclusive society, and the Second World War and mass eradication of the mentally ill made nations reevaluate the dangers of the hygienist’s propaganda.

The mental hygiene movement in India, as elsewhere, campaigned over the control and segregation of mental deficiency. Jonathan Toms has argued that the mental hygiene movement in Britain ‘continued to campaign on the “problem of mental deficiency” but it focused particularly on what it considered psychological causes of “social failure” in the wider population. This focus was strongly informed by psychodynamic thinking. An emphasis on “emotional adjustment” in the interests of adequate citizenship and social efficiency developed – with this construed in terms of mental health’.^129^ The question of citizenship and ‘national problems’ became acute in the era of anti-colonial struggle. Degeneration was linked to the poorer sections of society. Pacheco argued that ‘[h]ere is a fairly typical example of one among the poorer classes. The parents are poor, either or both of them have some neuropathic taint, in spite of which they have foolishly married. A child is born poor in health, mental and physical. Soon it is found to be backward and as such allowed to remain at home and grow at best it can in an environment where squalor, disease, crime and cruelty add to its sordid upbringing’.^130^ In other words, poverty breeds crime, disease, and mental deficiency as well. This fear of feeble-mindedness accounts for the fact that these children often ended up as adults in mental hospitals, where they were regarded as ‘burdensome’. The criminal lunatics were responsible for the hard labour that was necessary for the running of the asylums.^131^ The ‘idiots’ were not able to work and act as a labour force in the mental hospitals.

The advocacy for care of the feeble-minded often did not match day-to-day practices, as mental hospitals had no provisions to look after the so-called mentally deficient. Winifred Tarr, Secretary of the Red Cross Welfare Committee in India, remarked that the proposal for care and treatment in the Nagpur mental hospital was turned down. He noted, ‘[t]he running expenses for the unit of six children worked out at Rs. 100/- per month, the scheme was turned downed because the benefits to the state were not commensurate with the cost’.^132^ Historians working on medicine in South Asia have argued that the state played a limited role.^133^ The funds were retained for keeping checks on epidemics and other deadly diseases. Mental health was no longer regarded as a segment of the medical provision that needed any upgrade. There were two special institutions for the care of the mentally deficient. Kalidas Bhattacharyya was the principal of Lady Noyce School for deaf and dumb in New Delhi. He, in the statistical survey, discussed that ‘[t]here are only two schools for the feeble-minded children in India, both being in the province of Bengal. “[t]he Children’s house in Kurseong was founded by Miss S. de la Place in 1918, and the “Bodhana Niketan” formerly in Jhargram, Midnapur, now in Belgharia, 24-Parganas, was started by Girija Bhushan Mukherjee in 1933’.^134^ The Kurseong institution was especially for the European and Anglo-Indian population. The preservation of race and racial superiority was extremely significant for the Raj.^135^

The Bodhana Niketan was established on 1 July 1933 by Girija Bhushan Mukherjee who established a school for the care of feeble-minded. The *Bodhana Niketan* received funding from the government of Bengal. Mukherjee was a member of the Indian Association of Mental Hygiene and *Bodhana Niketan* had a formal association with the movement. The *Bodhana Niketan* had an established governing body and received support from the cultural elite of Bengal. Rabindranath Tagore named the institution as *Bodhana Niketan* or ‘House of enlightened’.^136^ Initial funding came from the Raja of Jhargram. Mukherjee’s motivation to establish institution for the feeble-minded came from the fact that his two sons suffered from mental deficiency.^137^ He was an advocate of the issue and pleaded ‘[t]he remedy lies in putting them in *special institutions*, where they have a world of their own with a peculiarly suited environment, and where their handicap casts no shadow, and where only those people take charge of them who take it as a mission in their lives’.^138^ Mukherjee validated the prevalent ideas about segregation and firmly believed that the feeble-minded should be segregated. He, like others, discussed the importance of enumeration to tackle the problem. The feeble-minded were considered to be a ‘social menace’ and the ‘national problem’. Mukherjee stated that ‘[T]he first thing that we give to these children is LOVE’.^139^ Nonetheless, he felt that the feeble-minded are ‘a source of constant embarrassment and considerable worry to their own parents even.’^140^

The feeble-minded aroused ‘shame’ and were regarded as ‘unfit’ in society. Indians internalised the ideas of embarrassment and regarded segregation as a possible solution to the ‘social menace’. This is not to argue that Indians were unaware of evolutionary ideas. Luzia Savary has worked on the conception of racism, heredity, and evolutionary ideas in Hindi and Urdu literature. She emphasised that the words ‘“race”, *Jati* and *nasl* reflect similar and yet different ways to group people that are based on a set of contested concepts of human difference’.^141^ There was a boom in vernacular literature about the correct ‘sexual practices’ in order to procreate a ‘better race’. This didactic literature on reproduction dealt with ‘fears’ and notions about ‘progress’ and what these ideas meant to these communities in the decisive times of anti-colonial struggles. Savary has argued that this reproductive literature on ‘the science of progeny’ had interesting parallels with ideas on ‘selected breeding’ or eugenics.^142^ She focusses on ‘*Santani-shastra*’s theory of hereditary transmission was based on the idea that parents could substantially influence the bodily and their intellectual features of their future offspring if they committed their “mental force”’.^143^ In other words, the consciousness or ‘mental force’ of the parents was most significant in the process of procreation. The parents, especially from the lower castes, who consumed alcohol had higher chances of having feeble-minded children.^144^ These views about alcohol and degeneration were widely accepted, but the consciousness of giving birth to a progeny that will be fair-skinned or more intelligent in some ways resonates with caste consciousness. The human consciousness is regarded as a significant force in Hinduism. ‘The mental’ and ‘physical’ collapsed into each other further as categories during the anti-colonial struggle. It has been argued elsewhere how ‘the power of mind’ emerged as a potent category to cure what nationalists regarded as a phase of ‘national impotency’.^145^ Physical weakness was a result of the loss of mental control over oneself, and as a result, mental weakness caused physical debility. The concepts underwent metamorphosis, adapting/filtering notions of self/other which were deeply linked to caste, gender, and communities, and, above all, *rashtra* or nation.^146^ The place of the feeble-minded in newly emerging nation-states like India was not created, and therefore no policies were formulated by the colonial state or the nationalists during the period.

## Conclusion

Epistemological growth in the fields of anthropology, phrenology, psychology, psychiatry, and neurology during the nineteenth century allowed psy-sciences to emerge and define all aspects of human life. From learning ability to mental measurement – the scaling, mapping, and naming of the mental – became a global concern. The twentieth century was an era of mapping the mind.^147^ The mental hygiene movement gave precedence to preservation, conservation, and observation of mental health. This movement expanded the notion of mental illness by including social issues such as alcoholism, delinquency, and syphilis.

The mental hygiene movement was a precursor to the mental health movement in India. Mental hygiene reemerged and became a part of the mental health movement in the Nehruvian era. The Indian Council for Mental Hygiene was re-established under the leadership of Keki Rustom Masani.^148^ In 1948, the Indian Council for Mental Hygiene became one of the founding members of the World Federation for Mental Health. Masani also opened several child guidance clinics in 1952.^149^ The second five-year plan and the establishment of the Indian National Committee for the World Mental Health Year (1960) emphasised the need for prevention and spreading awareness about mental health issues.^150^ In 1963, the mental health subcommittee placed mental hygiene as a mandatory ten-hour part of learning curriculum for the training of psychiatrists.^151^

This research has investigated an era when the mind sciences gained control and defined every aspect of the so-called ‘ideal citizen’. A novel taxonomy creating moralistic ideals, establishing rules, and a utopian vision formulated a cultural programme that attempted to preserve decaying imperialism and bourgeoning nationalism. The definition of ‘ideal citizenship’ was also invented in the colonies. This paper comprehends the influence of ideas such as degeneration, social Darwinism, and mental hygiene in colonies like India. Indian nationalism and the partition that followed in some ways show how *othering* had occurred at the popular level.

## Footnotes

1 ‘Rules for Mental Hygiene,’ *The Quarterly Bulletin of the Indian Association for Mental Hygiene*, 33 (1937), 5.

2 Moral management was the nineteenth century therapeutic approach of curing the insane with diet, routine, and practices of self-control. For more details, see Jonathan Toms, *Mental Hygiene and Psychiatry in Modern Britain* (Basingstoke, UK: Palgrave Macmillan, 2013).

3 The biological turn of psychiatry in the twentieth century gathered support from public health, allowing mental hygiene to emerge as the cultural movement.

4 Edited and Published by Lt. Col. Berkeley Hill. Printed by B. N. Chakrabarthy at Union Press.

5 See Carlo Ginzburg, *Cheese and the Worms* (Baltimore: John Hopkins University, 1992).

6 The Indian Association was affiliated to the National Council of Mental Hygiene. The Indian Mental Hygiene Movement like the British Model had limited state support and remained within the confines of elite groups promoting psychotherapy. This was much different from the American model which was more populist, promoted self improvement and had economic assistance from the state and private sectors.

7 Shilpi Rajpal, ‘Psychiatrists and Psychiatry in Late Colonial India.’ *The Indian Economic and Social History Review*, 55, 4 (2018), 545.

8 No systematic efforts were made by the colonial state or the burgeoning Indian National Movement to extend support or promote the mental hygiene movement in India. The movement remained limited to colonial officials or Indian elites who were interested in mental hygiene.

9 For detailed discussion see, Waltraud Ernst, Colonialism and Transnational Psychiatr*y: The Development of an Indian Mental Hospital in British India, c. 1925–1940*, 1st edn (New York: Anthem Press, 2013).

10 For details see, Berkeley Hill, ‘A Plea for the Inception of the Mental Hygiene Movement in India.’ *Indian Medical Gazette*, 58, 6 (1923), 242–244.

11 Shilpi Rajpal, ‘Psychiatrists and Psychiatry’, 544.

12 For more details see, Natalia Gerodetti, ‘Rational Subjects, Marriage Counselling and the Conundrums of Eugenics.’ *Studies in History and Philosophy of Biological and Biomedical Sciences*, 39, 2 (2008), 255–262.

13 Natalia Gerodetti, ‘Rational Subjects, Marriage Counselling and the Conundrums of Eugenics’, 256.

14

Mathew Thomson , ‘Mental Hygiene as an International Movement’, in Paul Weindling (ed.) International Health Organisations and Movements, 1918–1939 (Cambridge: Cambridge University Press, 1995), 283–284.

15 Gerald N. Grob, *Mental Illness and American Society, 1875–1940* (New Jersey: Princeton University Press, 1983), 148.

16 Gerald N. Grob, Mental Illness and American Society*, 1875–1940* (New Jersey: Princeton University Press, 1983), 151.

17
*Ibid.*, 298.

18

Christiane Hartnack , Psychoanalysis in Colonial India (New Delhi: Oxford University Press, 2001), 32 .

19 Ashis Nandy, *The Savage Freud and Other Essays in Possible and Retrievable Selves* (New Delhi:Oxford University Press, 2000), 97.

20 J. E. Dhunjibhoy was the superintendent in charge of the Indian Mental Hospital at Ranchi. There existed some amount of professional rivalry between Dhunjibhoy and Berkeley Hill who was the superintendent in charge of the European Mental Hospital at Ranchi. For more details on Dhunjibhoy, see, Waltraud Ernst , ‘The Indianization of Colonial Medicine: The Case of Psychiatry in Early-Twentieth-Century British India’, Journal of the History of Science, Technology and Medicine, 20, 4 (2012), 73 10.1007/s00048-012-0068-722555368.

21
*Ibid.*

22 Waltraud Ernst, *Mad Tales from the Raj: Colonial Psychiatry in South Asia, 1800–58* (New York: Anthem Press, 2010), 126.

23

James H Mills , ‘Reforming the Indian: Treatment Regimes in the Lunatic Asylums of British India, 1857–1880’. Indian Economic Social History Review, 36, 4 (1999), 407–429 21391370 10.1177/001946469903600401.

24

Nile Green , ‘Jack Sepoys and the Dervishes; Islam and the Indian Soldier in Princely India’. Journal of Royal Asiatic Society, 18, 1 (2008), 31–46 .

25 For details see, Nile Green, ‘Jack Sepoy and the Dervishes: Islam and the Indian Soldier in Princely India’, 31–46.

26

Waltraud Ernst . ‘Practising “Colonial” Or “Modern” Psychiatry in British India? Treatments at the Indian Mental Hospital at Ranchi, 1925–1940’ in Waltraud Ernst and Thomas Mueller (eds), Transnational Psychiatries: Social and Cultural Histories of Psychiatry in Comparative Perspective c. 1800–2000 (Newcastle: Cambridge Scholars Publishing, 2010), 80–115.

27 For details see, Alf Hiltebeitel, *Freud’s India: Sigmund Freud and India’s First Psychoanalyst Girindrasekhar Bose*, online edn (New York: Oxford Academic, 2018); Christiane Hartnack, *Psychoanalysis in Colonial India* (Oxford: Oxford University Press, 2001); T.G. Vaidyanathan and J.J. Kripal (eds), *Vishnu on Freud’s Desk: A Reader in Psychoanalysis and Hinduism* (Oxford: Oxford University Press, 1999); S. Akhtar and P. Tummala-Narra, ‘Psychoanalysis in India’, in Freud Along the Ganges: Psychoanalytic Reflections on the People and Culture of India. Edited by Salman Akhtar. New York: Other Press, 2005.

28
*The Quarterly Bulletin of the Indian Association for Mental Hygiene, Editorial, *13* *(1931) 29.**

29
*The Quarterly Bulletin of the Indian Association for Mental Hygiene, Editorial, *13* *(1931) 29.**

30
*The Quarterly Bulletin of the Indian Association for Mental Hygiene*, 13 (1931), 16–17.

31 The Young Men Christian’s Association. *The Quarterly Bulletin of the Indian Association for Mental Hygiene*, 13 (1931), 30.

32
*The Quarterly Bulletin of the Indian Association for Mental Hygiene*, Editorial, 13 (1931), 29.

33

S. C. Mitra , ‘History of the Psychology Department of the University of Calcutta’, Indian Journal of Psychology, XII, 3 (1937), 159 .

34
*Ibid.*

35
*The Quarterly Bulletin of the Indian Association for Mental Hygiene*, 31 (1936), 12.

36 For references on caste and communalism, see Charu Gupta, *Sexuality, Obscenity, Community: Women, Muslims and the Hindu Public in Colonial India* (New York: Palgrave, 2002), 69.

37
*The Quarterly Bulletin of the Indian Association for Mental Hygiene*, 26 (1935), 18.

38 An Extract from a Note on the Working of the Bengal After-Care Association (by kind permission of Mr H. Bivar, I.C.S., Session Judge, Murshidabad, Bengal), *The Quarterly Bulletin of the Indian Association of Mental Hygiene*, 11 (1931) 10.

39 Catriona llis, ‘Industrious, Truthful, Upright and Manly: Reforming Poor Young Men in the Borstal Schools of India in the 1920s and 1930s.’ *Gender & History*, 36, 3 (2024), 866.

40 An Extract from a Note on the Working of the Bengal After-Care Association (by kind permission of Mr H. Bivar, I.C.S., Session Judge, Murshidabad, Bengal), 11 (1931), 11.

41

Matthew Smith , Vicky Long , Oonagh Walsh , and Despo Kritsotaki , ‘Introduction’ in Despo Kritsotaki, Vicky Long , Matthew Smith (Ed.) Preventing Mental Illness: Past, Present and Future (Switzerland: Palgrave Macmillan, 2019), 17 .

42 The *Quarterly Bulletin of the Indian Association for Mental Hygiene*, 26 (1935), 20.

43

Norman J. Pacheco , Modern Methods in Psychiatry (Ranchi, Catholic Press 1935), 216 .

44
*The Quarterly Bulletin of the Indian Association for Mental Hygiene*, 26 (1935), 22.

45
*The Quarterly Bulletin of the Indian Association for Mental Hygiene*, 33 (1937), 1.

46 Discussion on Hill’s eccentric behavior is available in Waltraud Ernst, ‘The Indianization of Colonial Medicine: The Case of Psychiatry in Early-Twentieth-Century British India.’ *Naturwissenschaften, Technik Und Medizin*, 20, 2 (2012), 72.

47
*The Quarterly Bulletin of the Indian Association for Mental Hygiene*, 26 (1935), 1.

48
*The Quarterly Bulletin of the Indian Association for Mental Hygiene*, 33 (1937), 5.

49 Ernst, *op. cit.* (note 17), 71.

50
*The Quarterly Bulletin of the Indian Association for Mental Hygiene*, 33 (1937) 1.

51
*The Quarterly Bulletin of the Indian Association for Mental Hygiene*, 33 (1937) 2.

52

Shilpi Rajpal , Curing Madness? A Social and Cultural History of Insanity in North India (New Delhi; Oxford University Press, 2020), 212 .

53

Banarsi Das , ‘A Psychiatric Survey of Life’, Quarterly Bulletin of the Indian Association for Mental Hygiene, 35 (1937), 11 .

54
*Ibid*., 12.

55

P.G.G. Unnithan , ‘Mental Hygiene’, Quarterly Bulletin of the Indian Association for Mental Hygiene, 14 (1932), 11 .

56

Owen Berkeley Hill , ‘Parents and their Surrogates’, Quarterly Bulletin of the Indian Association for Mental Hygiene, 9 (1931), 1 .

57 A. Helen Boyle, ‘Family Life: Some Difficulties and Dangers’, *Quarterly Bulletin of the Indian Association of Mental Hygiene.* This lecture was originally delivered at the National Council for Mental Hygiene at the Royal Pavilion, Brighton, on 8 November 1934. It was summarized by the editor for *Quarterly Bulletin of the Indian Association for Mental Hygiene*, 28 (1935), 19.

58
*Ibid.*, 24.

59 Mrinalini Sinha, *The Spectres of Mother India; The Global Restructuring of an Empire* (Durham: Duke University Press, 2006), 2.

60
*Ibid*., 5–6.

61

Mrinalini Sinha , The Spectres of Mother India; The Global Restructuring of an Empire (Durham: Duke University Press, 2006), 1.

62
*Ibid.*, 2.

63
*Ibid.*, 4.

64
*Ibid.*

65
*Ibid.*, 46.

66

Ishita Pande , Sex, Law and the Politics of Age; Child Marriage in India, 1891–1937 (Cambridge; Cambridge University Press, 2020), 2 .

67 Owen Berkeley Hill, ‘Sex Hygiene’, *The Quarterly Bulletin of the Indian Association for Mental Hygiene*, 34 (1937), 14.

68

Owen Berkeley Hill , ‘Sex Hygiene’, The Quarterly Bulletin of the Indian Association for Mental Hygiene, 34 (1937), 13 .

69
*Ibid.*, 14.

70
*Ibid.*, 17.

71 Rajpal, *op. cit.* (note 45), 212.

72 For discussion on Freud and Masturbation, See George J. Makari , ‘Between Seduction and Libido: Sigmund Freud’s Masturbation Hypotheses and the Realignment of His Etiologic Thinking, 1897–1905’, Bulletin of the History of Medicine, 72, 4 (1998), 661–662 10.1353/bhm.1998.02169879122.

73 Rajpal, *op. cit.* (note 45), 206.

74

Berkeley Hill , *‘* Autoeroticism’, The Quarterly Bulletin of the Indian Association for Mental Hygiene, 27 (1935), 20 .

75
*Ibid.*, 23.

76
*Ibid.*

77
*Ibid.*, 24.

78 Makari, *op. cit.* (note 64), 661.

79 Charu Gupta, ‘Procreation and Pleasure: Writings of a Woman Ayurvedic Practitioner in Colonial North India. *Studies in History*, 21, 1 (2005), 33.

80 Rajpal, *op. cit.* (note 45), 207.

81 Pande, *op. cit.* (note 58), 192.

82

Ishita Pande , ‘Time for Sex: The Education of Desire and the Conduct of Childhood in Global/Hindu Sexology’ in Veronika Fuechtner , Douglas E. Haynes , and Ryan M. Jones (eds), A Global History of Sexual Science, 1880–1960 (California: University of California Press, 2018), 289 .

83

Charu Gupta , *Sexuality* *,* *O*bscenity, Community: Women, Muslims and the Hindu Public in Colonial India (New York: Palgrave, 2002), 69 .

84

Sarah Hodges , ‘South Asia Eugenic Pasts’, in Alison Bashford and Philipa Levine (eds), The Oxford Handbook of the History of Eugenics (London: Oxford University Press, 2010), 230 .

85
*Ibid.*

86
*Ibid.*

87

Erica Dyck , Facing Eugenics: Reproduction, Sterilization and the Politics of Choice (Toronto: University of Toronto Press: 2013), 5 .

88

Ian Robert Dowbiggin , Keeping America Sane: Psychiatry and Eugenics in the United States and Canada 1880–1940 (Ithaca and London: Cornell University Press, 2003), x .

89 Pande, *op. cit.* (note 58), 170.

90

Owen Berkeley Hill , ‘Some Problems and Crises of the Early Social Developments’, The Quarterly Bulletin of the Indian Association for Mental Hygiene, 35(1937), 4.

91 Ranjana Saha, ‘Motherhood on Display; The Child Welfare Exhibition in Colonial Calcutta 1920’, *Indian Economic and Social History Review*, 58. 2 (2021), 250.

92
*Ibid*.

93
*Ibid.*, 252.

94

Ruth Young , ‘The Mental Hygiene of the Preschool Child’, Quarterly Bulletin of the Indian Association for Mental Hygiene, 26 (1935) 5 .

95
*Ibid.*

96
*Ibid.*, 6

97

Rima D. Apple , Perfect Motherhood; Science and Childrearing in America (New Brunswick: Rutgers University Press 2006), 2 .

98

Berkeley Hill , ‘Parents and their Surrogates’, Quarterly Bulletin of the Indian Association for Mental Hygiene, 9 (1931), 5 .

99 Owen Berkeley Hill, ‘Some Problems and Crises of the Early Social Developments’, *The Quarterly Bulletin of the Indian Association for Mental Hygiene*, 35 (1937), 11. The exact term has been used to describe masturbation in children and children’s curiosity regarding sexuality.

100 For more details see, Caroline Vollans , Wise Words: How Susan Isaacs Changed Parenting (London: Routledge, 2017).

101

Owen Berkeley Hill , ‘Some Problems and Crises of the Early Social Developments’, The Quarterly Bulletin of the Indian Association for Mental Hygiene, 35 (1937), 4

102
*Ibid.*, 7.

103
*Ibid.*, 9.

104
*Ibid.*

105
*Ibid.*, 11

106
*Ibid.*, 11

107 Vollans, *op. cit.* (note 90), 3.

108 Hill, *op. cit.* (note 91), 3.

109
*Ibid.*, 13.

110

K. Clement Jones , ‘Psychology in its Relation to Child-life’, The Quarterly Bulletin of the Indian Association for Mental Hygiene, 32 (1936), 21 .

111

H. Stedman , ‘The Boy who Breaks all the Rules’, The Quarterly Bulletin of the Indian Association for Mental Hygiene, 5 (1930), 23 .

112

Kathleen W. Jones , Taming the Troublesome Child: American Families, Child Guidance, and the Limits of Psychiatric Authority (Cambridge: Harvard University Press, 1999), 8.

113

H. Stedman , ‘The Boy Who Breaks All the Rules’, Quarterly Bulletin of the Indian Association for Mental Hygiene, 5 (1930) 23 .

114 Jones, *op. cit.* (note 102), 14.

115

P.G.G. Unnithan , ‘Mental Hygiene’, Quarterly Bulletin of the Indian Association for Mental Hygiene, 14 (1932), 11 .

116

Laura D. Hirshbein , ‘Assessing the Conduct of Juveniles: Diagnosis and Delinquency, 1900–2013’, Medical History, 65, 4 (2021), 348 .

117 ‘Report for the Child Guidance Clinic for the Sir Dorabji Tata Graduate School of Social Work’, *Indian Journal of Social Work*, 1, 1 (1940), 101–102.

118

John Stewart , ‘“The Dangerous Age of Childhood”: Child Guidance and the “Normal” Child in Great Britain, 1920–1950’, Paedagogica Historica: International Journal of the History of Education, 47, 6 (2011), 786 .

119

J.N.J. Pacheco , ‘A Plea for the Control of the Mentally Defective Population in India’, The Quarterly Bulletin of the Indian Association for Mental Hygiene, 7 (1930), 3 .

120
*Ibid.*

121

Mark Jackson , The Borderland of Imbecility: Medicine, Society and the Fabrication of the Feeble Mind in Late Victorian and Edwardian England (Manchester: Manchester University Press, 2000), 3.

122 J. N. J. Pacheco, ‘A Plea for the Control of the Mentally Defective Population in India’, *The Quarterly Bulletin of the Indian Association for Mental Hygiene*, 7 (1930), 2.

123
*Ibid.*

124 Kalidas Bhattacharyya, *A Statistical Survey of the Present Educational Condition of the* *Infirm*: *the Deaf* *-Mutes* *, the Blind, and the Feebleminded – in* *India* (Calcutta: Calcutta University Press, 1938), 13.

125 The colony system was regarded as a solution to the problem of feebleminded. The idea behind the colony system was that would help in segregation and in further controlling reproduction of ‘mental defectives’ who were regarded as responsible for economic impoverishment and causing the national deficiency. The colony system was inspired by the Gheel-like German village or a villa design asylum at Toleda, Ohio.

126

Mathew Thomson , ‘Sterilization, Segregation and Community Care. Ideology and Solutions to the Problems of Mental Deficiency in Inter-War Britain’, History of Psychiatry, 3, 12 (1992), 494 10.1177/0957154X920030120511645803.

127

Owen A. R. Berkeley-Hill , Collected Papers (The Book Company, Ltd.: College Square, Calcutta, 1933), 244 .

128
*Ibid.*

129

Jonathan Toms , ‘Citizenship and Learning Disabled People: The Mental Health Charity MIND’s 1970s Campaign in Historical Context’, Medical History, 61, 4 (2017), 484 10.1017/mdh.2017.55PMC562960628901871.

130

J. N. J. Pacheco , ‘A Plea for the Control of the Mentally Defective Population in India’, Quarterly Bulletin of the Indian Association for Mental Hygiene, 7 (1930) 5 .

131 Rajpal, *op. cit.* (note 45), 113

132

Winifred Tarr , ‘Proposal for Care and Treatment of Feeble-Minded Children in a Mental Hospital’, Quarterly Bulletin of the Indian Association for Mental Hygiene, 9 (1931), 11 .

133

Biswamoy Pati and Mark Harrison , ‘Social History of Health and Medicine: Colonial India’, in Biswamoy Pati and Mark Harrison (eds), The Social History of Health and Medicine in Colonial India (London: Routledge, 2009), 6.

134 Bhattacharyya, *op. cit.* (note 115), 48–49.

135

Waltraud Ernst , Mad Tales from the Raj: The European Insane in British India, 1800–1858 (London: Routledge, 1991).

136
*The Quarterly Bulletin of the Indian Association for Mental Hygiene*, 22 (1934), 5.

137 ‘The Bodhana Samiti or the Association for the Care and Control of the Feeble-Minded-First Annual Report’, *The Quarterly Bulletin of the Indian Association for Mental Hygiene*, 20 (1933), 15.

138

Girija Bhushan Mukherjee , ‘Bodhana Samiti its Work with Feeble-Minded Children’, The Quarterly Bulletin of the Indian Association for Mental Hygiene, 22 (1934), 5 .

139
*Ibid.*, 5.

140
*Ibid.*, 7.

141

Luzia Savary , Evolution, Race and Public Spheres in India: Vernacular Concepts and Sciences, 1860–1930 (London; Routledge Studies in South Asian History, 2019), 5 .

142
*Ibid.*, 11.

143
*Ibid.*, 102.

144
*Ibid.*, 103.

145 Rajpal, *op. cit*. (note 45), 227.

146
*Ibid.*

147 For more details see, Erik Linstrum , Ruling Minds: Psychology in the British Empire (Cambridge, MA: Harvard University Press, 2016).

148
*Ibid.* The historical research on psychiatry in postcolonial India is scarce. The information available on Keki Masani and the formation of Indian Council of Mental Hygiene is also limited.

149
Dr. Kaikhushru Rustom Masani , ‘Icons of Indian Psychiatry’, Indian Journal of Psychiatry 52 (2), January 2010, 161–162 .

150

Alok Sarin and Sanjiv Jain , ‘Mapping Difficult Terrains: The Writing of Policy on Mental Health’, in R. White, S. Jain, D.Orr and U. Read (eds), The Palgrave Handbook of Sociocultural Perspectives on Global Mental Health (Palgrave Macmillan, 2017), 710 .

151 The Mental Health Sub-Committee of 1963, The Government of India. The Agra Mental Hospital.

