# The mental hygiene movement: the birth of global mental health in India – CORRIGENDUM

**DOI:** 10.1017/mdh.2025.10050

**Published:** 2026-01

**Authors:** Shilpi Rajpal

**Keywords:** Mental hygiene, Decolonization, Global mental health movements, Eugenics, Motherhood, Degeneration

The Author apologises for omitting an acknowledgement of Danish National Research Foundation grant DNRF171. The Acknowledgements section should therefore read as follows:

## References

[r1] Rajpal Shilpi, The mental hygiene movement: the birth of global mental health in India. Medical History. Published by Cambridge University Press, 5 December 2025. doi:10.1017/mdh.2025.10035

